# Supplementary material for: CEPS: An Open Access MATLAB Graphical User Interface (GUI) for the Analysis of Complexity and Entropy in Physiological Signals
Source: Entropy (Basel). 2021 Mar 8;23(3):321. doi: 10.3390/e23030321 (PMC7998823; doi:10.3390/e23030321)
Supplement: Supplementary file 1 [file entropy-23-00321-s001.pdf]

CEPS. An open-access MATLAB graphical user interface (GUI) for the analysis of Complexity and Entropy in Physiological Signals

David Mayor, Deepak Panday, Harikala Kandel, Tony Steffert and Duncan Banks

Supplementary material

SM1. A brief review of studies comparing nonlinear measures of complexity or entropy with traditional linear measures

Rather than trying to review all studies on each nonlinear measure that we considered using in CEPS, studies were located in PubMed using the name of each measure (e.g. 'Sample entropy') together with the word 'better' or 'worse'. This reduced the number of studies to something more manageable. The Abstracts – but not the studies themselves – were then examined for comparisons between complexity/entropy and linear or traditional measures, and counts were taken of when the former or the latter were found more useful by the authors of those studies. In many studies, this was not possible, usually because the focus of the study was not on the particular comparison we were interested in (the words 'better' or 'worse' being applied to other comparisons).

The results of this review are shown in tabular form below (Tables S1 and S2). The first column indicates the acronym for the measure in question, the second column the number of studies including the word 'better' (followed by the number including the word 'worse' in parentheses). The third column shows the type of data being investigated, the fourth the first author of the study and year of publication, in date order. In the fifth column, 'y' indicates that nonlinear measures were found in some sense 'better' than the linear or traditional measures with which they were compared (shown in the final column), 'n' that they were 'worse', and 'd' that the measures appeared to quantify different underlying dynamics and so were not directly comparable.

Table S1. Review results – complexity measures.

Measure	*N*	System	Study	Nonlinear better	Comparator
**HFD**	9 (1)	iv ultrasound	Santos Filho *et al.* 2008 [154]	y	Correlation
		Intracranial	Jouny & Bergey 2012 [155]	y	Band power
		Retina	Aliahmad *et al.* 2014 [156]	y	'Other'
		EEG	Kawe *et al.* 2019 [157]	y	Alpha asymmetry
**KFD**	1 (1)	[n/a]			
**Allan Factor**	1 (0)	HRV	Pawlak-Buś *et al.* 2005 [158]	y	Standard method
**D_2**	30 (1)	HRV	Skinner *et al.* 1993 [159]	y	Standard deviation
		EEG	Pritchard *et al.* 1994 [160]	y	Standard linear

		Myoelectric	Nieminen & Takala 1996 [161]	y	Random stochastic
		EEG	Stam *et al*. 1996 [162]	y + Pwr[1]	Power/reactivity
		EEG (sleep stage)	Fell *et al*. 1996 [163]	y / n	Spectral measures
		EEG	Grözinger *et al*. 2001 [164]	n	Spectral powers
		ECG, EEG	Balli & Palaniappan 2010 [165]	n	Linear AR[2] model
		Voice	Gao & Hu 2012 [166]	y	Traditional linear
		EEG	Eagleman *et al*. 2018 [167]	y	Traditional spectral
**H**	30 (0)	fMRI	Maxim *et al*. 2005 [168]	y	AR model (order 1)
		HRV	Yum *et al*. 2008 [169]	y	Spectral indices
		HRV	Yeh *et al*. 2010 [170]	n	Empirical mode decomposition;
		ECG, EEG	Balli & Palaniappan 2010 [165]	n	Linear AR model
**DFA**	44 (4)	HRV	Mäkikallio *et al*. 1998 [171]	y	Conventional HRV measures
		HRV	Hotta *et al*. 2005 [172]	y	Traditional linear
		fMRI	Lee *et al*. 2008 [173]	y	Deconvolution
		HRV	Heitmann *et al*. 2011 [174]	y	Traditional linear
		HRV	Jiang & Wu 2011 [175]	y	Spectral indices
		HRV (sleep stage)	Tseng *et al*. 2013 [176]	n	Spectral power ratio
		HRV	Téllez *et al*. 2014 [177]	n	Discrete wavelet transform
		Respiration	Mumtaz *et al*. 2015 [178]	y	Logistical regression
		EEG	Castiglioni *et al*. 2019 [179]	y	Traditional indices
		HRV	Tsai et a l. 2019 [180]	y	Traditional parameters
**LLE**	54 (2)	EEG (sleep stage)	Fell *et al*. 1996 [163]	y / n	Spectral measures
		EEG	Grözinger *et al*. 2001 [164]	n	Spectral powers
		Respiration	Yeragani *et al*. 2002 [181]	y	Traditional time domain
		EEG	Sackellares *et al*. 2006 [182]	y	Naïve prediction schemes
		EEG	Khoshnoud *et al*. 2018 [183]	y	Band power features
**RQA**	33 (0)	HRV	Javorka *et al*. 2008 [184]	d	Linear parameters
		HRV	Mohebbi *et al*. 2011 [185]	y	Other existing approaches
		Posturography	Ferrufino *et al*. 2011 [186]	y	Classical statistics
		Respiration	Terrill *et al*. 2012 [187]	y	Time-series & spectral anal.

		Emotional valence	Lichtwarck-A *et al.* 2012 [188]	y	Valence
		Surface EMG	Ito & Hotta 2012 [189]	y	Traditional frequency analysis
		HRV	Frondelius *et al.* 2015 [190]	d	Linear (time and freq. domain)
**PP**	24 (1)	HRV	Heitmann *et al.* 2011 [174]	y	Linear (time and freq. domain)
		EEG	Brignol *et al.* 2013 [191]	y	Spectral band power
		HRV	Shi *et al.* 2018 [192]	y	Frequency domain
		HRV	Farah *et al.* 2018 [193]	n	Frequency domain
		HRV	Byun *et al.* 2019 [194]	y	Linear indices
**LZC**	18 (0)	EEG	Zhang *et al.* 2001 [195]	y	Median frequency
		EEG	Huang *et al.* 2003 [196]	y	Median frequency, Spectral edge

1. Measure combined with Power better than Power alone; 2. AR: Autoregressive.

Table S2. Review results – entropy measures.

Measure	*N*	System	Study	Nonlinear better	Comparison
**SE**	69 (1)	EEG bicoherence	Li *et al.* 2011 [197]	y	Traditional FFT bicoherence
		Image denoising	Fathi & Naghsh-Nilchi 2012 [198]	y	Standard denoising
		Temperature	Papaioannou *et al.* 2013 [199]	y	Sequential organ failure
		Gene expression	Wang *et al.* 2014 [200]	d	Differential Coefficient of Variation
		Image features	Yang *et al.* 2014 [201]	y	SMACC
		HIV-1 identification	Wu *et al.* 2015 [202]	y	prior viral diversity markers
		Peanut allergens	Johnson *et al.* 2016 [203]	y	Standard search algorithms
		Brain mapping	Delic *et al.* 2016 [204]	y	Fractional anisotropy
		Water contamination	Khosravi *et al.* 2018 [205]	y	Weights-of-Evidence
		Sedimentation	Zhu & Peng 2019 [206]	y	Deterministic models
		MRI (DTI)	Liang *et al.* 2019 [207]	y	Traditional methods
**RE**	9 (1)	Mechanomyography	Torres *et al.* 2008 [208]	y	Other (linear) parameters
		EEG	Choi 2015 [209]	y	[Unclear from Abstract]
**M-E**	1 (0)	[n/a]			
**TE**	6 (0)	PET	Gao *et al.* 2013 [210]	y	Traditional methods
		Temperature	Papaioannou 2013 [199]	y	Sequential organ failure
		MRI	Zhang *et al.* 2015 [211]	y	Discrete wavelet transform

		EEG	Lazar *et al.* 2018 [212]	y	Bayes, Normal and Vis shrink
**KSE**	4 (0)	[n/a]			
**PE**	29 (0)	EEG	Silva *et al.* 2011 [213]	y / n	Spectral params / electromyography
		Accelerometer data	Ihlen *et al.* 2016 [214]	y	35 conventional gait features
		STABO	Aydın *et al.* 2019 [215]	y + SVM	Spectral params + naive Bayes
**CE/CCE**	10 (0)	Cardiac CT	Zhuang *et al.* 2015 [216]	y	Conventional schemes
**ApEn**	100 (2)	EEG	Bruhn *et al.* 2000 [217]	y	Median frequency
		Respiration	Yeragani *et al.* 2002 [181]	y	CV of tidal volume
		EEG	Bruhn 2003 [218]	y (nonsig)	Bispectral index, SEF95
		HRV	Gonçalves *et al.* 2007 [219]	d	Linear spectral indices
		ECG, EEG	Balli & Palaniappan 2010 [165]	n	Linear AR model
		Mechanomyography	Sarlabous *et al.* 2010 [220]	y	RMS amplitude
		EEG	López-Cuevas *et al.* 2013 [221]	y + ANN	Existing algorithms
		EEG	Hsu 2015 [222]	y	Conventional measures
		HRV	Choi & Hoh 2015 [223]	y	Conventional parameters
		Centre of pressure	Williams *et al.* 2016 [224]	y	RMS
		HRV	Kabbach *et al.* 2017 [225]	d	Linear measures
		Water quality	Huang *et al.* 2017 [226]	y	AR model, sequential probability
		EEG	Khoshnoud *et al.* 2018 [183]	y	Band power features
		Motion analysis	Zia *et al.* 2018 [227]	y	Prior state-of-the-art methods
		Muscle fatigue	Du *et al.* 2018 [228]	y	Power & frequency measures
		EEG	Chow *et al.* 2019 [229]	y	EEG Theta/beta ratio
		fMRI	Nan *et al.* 2019 [230]	y	Regional homogeneity
		fMRI	Zhang *et al.* 2020 [231]	y	Functional connectivity
		EEG	Saeedi *et al.* 2020 [232]	y	Frequency-based features
**SampEn**	120 (4)	EEG	Ke *et al.* 2014 [233]	y	EEG power ratio
		HRV	Gonçalves *et al.* 2007 [219]	d	Linear spectral indices
		Base lung sound	Albuerne-S *et al.* 2008 [234]	y	Spectral percentiles
		EMG	Zhou & Zhang 2014 [235]	y	Conventional methods
		HRV	Choi & Hoh 2015 [223]	y	Conventional methods
		Physical activity	Aktaruzzaman *et al.* 2015 [236]	y	AR coefficients
		Denoising	Shen *et al.* 2016 [237]	y	Traditional methods
		Local field potential	Zare *et al.* 2020 [238]	n	LFP power
		EEG	Saeedi *et al.* 2020 [232]	y	Frequency-based features

**CosEn**	0 (0)	[n/a]			
**QSE**	0 (0)	[n/a]			
**MSE**	43 (4)	Pulse wave velocity	Wu *et al.* 2011 [239]	y	Pulse wave velocity
		MEG	Chu *et al.* 2015 [240]	y	Rel power in some bands
		Postural sway	Zhou *et al.* 2017 [241]	y	Traditional sway metrics
		HRV	Tsai *et al.* 2019 [180]	y	Traditional HRV parameters
		HRV	Lin *et al.* 2016 [242]	y	SDNN, pNN20 (HRV parameters)
**FE**	23 (0)	EEG	Lazar *et al.* 2016 [243]	y	Traditional complex wavelet
**DistEn**	7 (0)	[n/a]			
**DE**	3 (0)	[n/a]			
**SlopeEn**	0 (0)	[n/a]			
**BE**	0 (0)	[n/a]			
**PhEn**	1 (0)	[n/a]			
**DnEn**	1 (0)	[n/a]			
**CompressionEn**	2 (0)	HRV	Heitmann *et al.* 2011 [174]	y	Time and frequency domain
**SpEn**	37 (5)	EEG	Grözinger *et al.* 2001 [164]	n	Spectral power
		EEG (sleep stage)	Fell *et al.* 1996 [163]	y / n	Spectral measures
		EEG	Martorano *et al.* 2006 [244]	y	Heart rate, mean arterial pressure
		EEG	Kim *et al.* 2012 [245]	n	Bispectral index
		EEG	Tiefenthaler *et al.* 2018 [246]	y	Bispectral index
**DiffEn**	7 (0)	Heart motion	Punithakumar *et al.* 2010, 2013 [247, 248]	y	Other recent methods

+: Combination of nonlinear and another method better or worse than comparator; ANN: Artificial neural networks; DTI: diffusion tensor imaging; iv: intravenous; LFP: Local field potential; SMACC: sequential maximum angle convex cone; STABO: single trial auditory brain oscillations; SVM: support vector machine.

In total, without deduplication, 716 studies were found for these measures in which the word ‘better’ occurred, and 27 containing the word ‘worse’ (suggesting that researchers much prefer to make positive rather than negative claims about their findings).

Of these studies (deduplicated), 83 (around 11%) found that nonlinear measures were more useful than linear or conventional measures in terms of their study objectives, and only 10 (around 1.3%) that they were less useful. Five studies mentioned, explicitly or implicitly, that the nonlinear and other measures used appeared to quantify different underlying dynamics so were not directly comparable.

Many studies could not be categorised in this way, as they used methods such as support vector machine classification or AdaBoost to assess the usefulness of *combinations* of measures, or compared nonlinear measures amongst themselves.

Because of the growing popularity of these combination methods, it was expected that there might be fewer studies comparing nonlinear and linear methods in recent years, but this was not found to be the case (Figure S1).

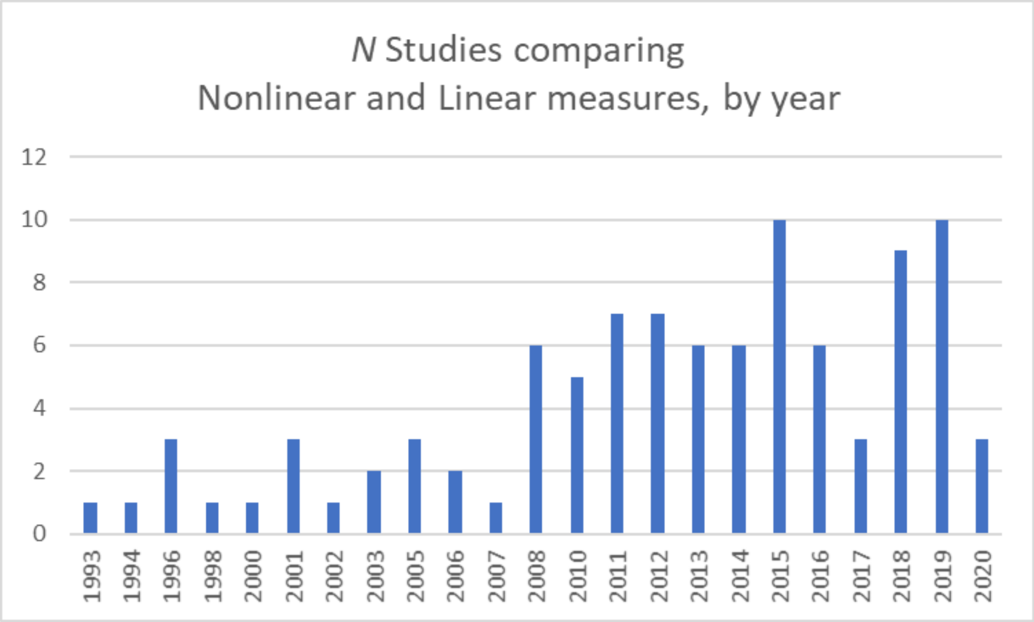

Figure S1. Incidence of studies comparing nonlinear and linear/conventional measures, over time.

SM2. Names of researchers who have contributed most papers on each complexity and entropy measure

Table C1. Numbers of researchers who have authored papers on complexity measures, with names of up to six authors who have written the most papers on each measure (more or less than six if numbers tied) and how many they have written, together with their institutional affiliations. Mean and standard deviation of the number of papers per author for each measure are also shown.

Measure (*N* authors[a])	Authors	1st mention	Institution[b]	Mean (SD)
**HFD** (425)	Tarmo Lipping (7)	2004	Tampere U of Technology	1.28 (0.75)
	Aleksandar Kalauzi (6)	2005	U of Belgrade	
	Sladjana Spasić (5)			
	Maie Bachmann, Hiie Hinrikus, Jaanus Lass (all 5)	2005<br>2011	Tallinn U of Technology	
**KFD** (110)	Hojjat Adeli (4)	2010	Amirkabir U of Technology, Tehran	1.05 (0.27)
	Amir Adeli (3)	2010		
	Mehran Ahmadlou (3)	2010		
**AF** (64)	Susan Barman (4)	2004	Michigan State U	1.27 (0.68)
	Gerard Gebber (4)	2004		
	Paul Fadel (3)	2004		
	Malvin Teich (3)	1996	Boston U	
**D_2** (2,140)	Jack J Jiang (31)	2003	U of Wisconsin	1.40 (1.29)
	Yu Zhang (23)	2003		
	James E Skinner (18)	1991	Baylor College of Medicine[c]	
	Cornelis J Stam (16)	1994	VU Medical Centre, Amsterdam	
	Joachim Röschke (12)	1991	U of Mainz	
**H** (1,410)	U Rajendra Acharya (9)	2005	Singapore U of Social Sciences	1.27 (0.75)
	Edward T Bullmore (9)	2001	U of Cambridge	
	John Suckling (9)	2001		
	Hans J Herrmann (8)	2011		

			Universidade Federal do Ceará	
**DFA** (3,500)	Chung-Kang Peng (19)	1995	U of Harvard	1.33 (1.07)
	H Eugene Stanley (19)	1995	Boston U	
	Ary L Goldberger (18)	1995	Beth Israel Hospital, Boston	
	Shlomo Havlin (15)	1995	Boston U	
	Heikki Huikuri (13)	1999	U of Oulu	
**LLE** (1,470)	Nick Stergiou (25)	2003	U of Nebraska	1.36 (1.14)
	Vikram Yeragani (12)	2001	Wayne State U	
	U Rajendra Acharya (9)	2005	Singapore U of Social Sciences	
	Thomas Similowski (9)	2006	Sorbonne Universités	
	Shane R Wurdeman (9)	2013	U of Nebraska	
**RQA** (1,200)	Alessandro Giuliani (25)	1998	National Institute of Health, Rome	1.47 (1.56)
	Michael A Riley (20)	1999	U of Cincinnati	
	Charles L Webber Jr (16)	1997	Loyola U	
	Joseph P Zbilut (16)	1998	Rush U	
**PP** (1,290)	Andreas Voss (19)	2007	Ernst-Abbe-Hochschule Jena	1.37 (1.30)
	Marimuthu Palaniswami (16)	2001	U of Melbourne	
	Luiz Vanderlei (16)	2010	UNESP, São Paulo	
	Ahsan Khandoker (15)	2008	U of Melbourne	
	Heiiki Huikuri (12)	1996	U of Oulu	
	Chandan Karmakar (11)	2008	U of Melbourne	
**LZC** (720)	Roberto Hornero (25)	2005	Universidad de Valladolid (all)	2.33 (3.09)
	Daniel Abásolo (17)	2006		

	Carlos Gómez (15)	2005		

a. Numbers are approximate, because of spelling, forename variations and differences in punctuation; b. Where authors are affiliated to more than one institution, only one of these is shown (selected according to the author's presumed country of origin); c. Then Vicor Technologies, Inc., Boca Raton, FL.

Deduplicating names, a total of approximately 10,200 individuals have authored papers on these complexity measures, with most authors writing on DFA and D_2, fewest writing on the two measures of fractal dimension and the Allan factor. Although fewer papers were published on LZC than several other measures, on average more papers were published on this topic by single authors than on any other topic, and fewest by single authors on Katz's fractal dimension.

Table C2. Numbers of researchers who have authored papers on Symbolic Dynamics, with names of up to six authors who have written the most papers on each measure (more or less than six if numbers tied) and how many they have written, together with their institutional affiliations. Mean and standard deviation of the number of papers per author for each measure are also shown.

**Measure (*N* authors)**	**Authors**	**1st mention**	**Institution**	**Mean (SD)**
**SymDyn** (895)	Andreas Voss (55)<br>Mathias Baumert (23)<br>Jürgen Kurths (17)<br>Niels Wessel (17)	1995<br>2002<br>1995<br>1995	U of Applied Sciences, Jena<br>Universität Potsdam<br>Humboldt-Universität zu Berlin	1.63 (2.50)

Table C3. Numbers of researchers who have authored papers on entropies, with names of up to six authors who have written the most papers on each measure (more or less than six if numbers tied) and how many they have written, together with their institutional affiliations. Mean and standard deviation of the number of papers per author for each measure are also shown.

**Measure (*N* authors[a])**	**Authors**	**1st mention**	**Institution[b]**	**Mean (SD)**
**SE**	Shubin Liu (18)	2007	U of North Carolina	1.17 (0.79)

(3,700)	Ki H Chon (15)	2009	U of Connecticut	
	Yi Zhang (11)	2009	Hebei U of Science and Technology	
	Jürgen Bajorath (11)	2000	Rheinische Friedrich-Wilhelms-Universität Bonn	
	David McManus (11)	2012	U of Massachusetts	
	Alberto Porta (11)	2001	U of Milan	
**AE & EoE** (3)	Chang Francis Hsu (3)<br>Long Hsu (3), Sien Chi (3)	2017	National Chiao Tung U (Hsinchu)	
**T-E**	Ahsan Khandoker (10)	2010	U of Melbourne	
(24)	Herbert Jelinek (8)		Charles Sturt U	
	Toshio Moritani (6)	1997	Kyoto U	
	Eiichi Oida (5)			
**RE**	Shubin Liu (6)	2016	Hunan Normal U	1.23 (0.63)
(430)	Chunying Rong (4)	2017		
	David Cornforth (4)	2013	U of Newcastle	
	Michael Hughes (4)	2009	Washington U	
	Jon Marsh (4)	2009		
	Kirk Wallace (4)	2009		
	Samuel Wickline (4)	2009		
	John McCarthy (4)	2010		
	Herbert Jelinek (4)	2013	Charles Sturt U	
**M-E** (73)	Jianhua Zhang (2)	2015	East China U of Science and Technology	1.01 (0.12)
**TE** (290)	Ervin Lenzi (6)	2001	U Estadual de Ponta Grossa	1.18 (0.62)
	Sumiyoshi Abe (5)	2002	U of Tsukuba	
	YuDong Zhang (5)	2008	Southeast U	
**KSE**	Jay Robert Dorfman (4)	1995	U of Maryland	1.18 (0.47)
(330)	Henk van Beijeren (4)	2001	Utrecht U	

**PE** (810)	Xiaoli Li (20)	2007	Beijing Normal U	1.40 (1.26)
	James Sleigh (13)	2008	Waikato Hospital	
	Denis Jordan (10)	2008	Technical U Munich	
	Zhenhu Liang (10)	2010	Yanshan U	
**CE/CCE** (540)	Alberto Porta (38)	1998	U of Milan	1.46 (2.14)
	Nicola Montano (17)			
	Luca Faes (16)	2010	U of Trento	
	Vlasta Bari (11)	2011	Politecnico di Milano	
	Eleonora Tobaldini (10)	2007	U of Milan	
**ApEn** (3,500)	Johannes Veldhuis (170)	1994	U of Virginia	1.70 (3.67)
	Steven Pincus (74)	1991	Yale U	
	Ferdinand Roelfsma (60)	1996	Leiden U	
	Ali Iranmanesh (40)	1996	Salem Veterans Affairs Medical Center	
**SampEn** (3,310)	Roberto Hornero (23)	2006	U of Valladolid	1.55 (1.55)
	José Joaquín Rieta (21)	2007	U Politécnica de Valencia	
	Raúl Alcaraz (20)	2007	U of Castilla - La Mancha	
	Pascal Madeleine (19)	2009	Aalborg U	
	Xin Li (17)		Yanshan U	
	Chengyu Liu (16)	2013	Shandong U	
	Jiann-Shing Shieh (14)	2014	Yuan Ze U	
	David Cuesta-Frau (13)	2007	U Politècnica de València	
**CosEn** (47)	All authors 1 paper only	2011	n/a	1.00 (1.00)
**QSE** (14)	Nina Burtchen (2)	2017	U of Freiburg	1.29 (0.47)
	William Fifer (2)		Columbia U	
	Maristella Lucchini (2)		Politecnico di Milano	

	Maria Signorini (2)			
**fSampEn**<br>(9)	Raimon Jané<br>Abel Torres<br>Leonardo Sarlabous<br>Luis Estrada	2016<br>2016<br>2016<br><br>2016	Universitat Politècnica de Catalunya	4.27 (2.57)
**MSE**<br>(1,368)	Chung-Kang Peng (30)<br>Madalena Costa (24)<br>Ary Goldberger (22)<br>Men-Tzung Lo (23)	2002<br>2002<br>2002<br>2011	Harvard U<br>Beth Israel Deaconess Medical Center, Boston<br>Taipei Veterans General Hospital	1.63 (1.84)
**FE**<br>(441)	Chengyu Liu (11)<br>U Rajendra Acharya (5)<br><br>Alberto Fernández (5)<br><br>Jianfeng Hu (5)<br><br>Peng Li (5)	2013<br>2016<br><br>2013<br><br>2017<br><br><br>2013	Southeast U<br>Singapore U of Social Sciences<br>U Complutense de Madrid<br>Jiangxi U of Technology<br>Shandong U	1.26 (0.81)
**DE**<br>(34)	Javier Escudero (4)<br>Hamed Azami (3)	2016	Edinburgh U	1.21 (0.64)
**SlopeEn**<br>(0)	David Cuestra-Frau (0)	[2019]	U Politècnica de València	n/a
**BE**<br>(5)	George Manis (1)<br>Roberto Sassi (1)<br>David Cuestra-Frau (1)	2017<br>2017<br>2019	U of IoanninaU degli Studi di Milano<br>Universitat Politècnica de València	1.00 (0.00)

**PhEn** (2)	Ashish Rohila (1)<br>Ambalika Sharma (1)	2019	Indian Institute of Technology	1.00 (0.00)
**DisteEn**[c] (28)	Peng Li (10)<br>Chandan Karmakar (8)<br>Marimuthu Palaniswami (7)	2013<br>2008<br>2001	Shandong U<br>U of Melbourne	1.29 (0.78)
**DnEn** (109)	Huijie Yang (8)<br>Paolo Grigolini (7)<br>Changgui Gu (3)<br>Nicola Scafetta (3)	2004<br>2002<br>2013<br>2002	U of Shanghai for Science and Technology<br>U of North Texas<br>U of Shanghai for Science and Technology<br>Duke U	1.33 (1.03)
**SpEn** (1,118)	Hornero Roberto (17)<br>Jesús Poza (13)<br>James Sleigh (11)<br>Alberto Fernandez (10)<br>Javier Escudero (8)	2006<br>2006<br>2001<br>2007<br>2007	U of Valladolid<br><br>Waikato Hospital<br>U Complutense de Madrid<br>Edinburgh U	1.33 (1.06)
**DiffEn** (208)	5 authors from 2 research groups were mentioned 5 times each:<br>Kumaradevan Punithakumar, Ismail Ben Ayed, Ali Islam<br>NA Kampanis<br>PG Katonis	 2009<br>2009<br>2009<br>2009	 GE Healthcare, London, ON<br>Technological and Educational Institute, Crete	1.38 (0.89)

a. Numbers are approximate, because of spelling, forename variations and differences in punctuation;

b. Where authors are affiliated to more than one institution, only one of these is shown (selected according to the author's presumed country of origin);
c. Quite a number of the 'Distribution entropy' studies listed in PubMed refer to methods other than those of Li *et al.*

Deduplicating names, a total of approximately 12,400 individuals have authored papers on these entropy measures, with most authors writing on SE and ApEn, fewest writing on new measures such as BE and PhEn. On average, more papers were published on ApEn by single authors than on any other entropy measure, and fewest by single authors on CosEn, BE and PhEn.

In total, around 20,100 individuals authored the papers on complexity or entropy measures that were located using PubMed on 6 August 2020.

SM3. Software packages for physiological data analysis, with primary references, showing operating system and platform used, which are GUIs, the main data types and formats for which the software can be used, complexity and entropy measures implemented, and numbers of Google Scholar and SCOPUS citations of the software. Citation numbers are approximate, and grouped as follows: <10 *; 10-100 **; 100-500 ***; 500-2,500 ****; 2,500-5,000 *****; >5,000 ******. Free or open-source software is indicated by '[F]' in the GUI column (or by '[f]' if supported only in a subscription version of the software), and if batch processing is known to be supported, this is indicated by '[B]' in the 'Format' column (or by '[b]' if supported only in a subscription version). Information on pre-processing methods available was also gathered, but is not included here. Note that it was not possible to locate all relevant details for every package. This Table is based in part on the useful summary by the creators of PyBioS [24].

Name	Platform	OS	GUI	Main purpose	Format	Complexity	Entropy	GoogleScholar	SCOPUS
**Acq-Knowledge** [249]			yes	?	product-specific			***	*
**aHRV** (Nevrokard) [25]			yes	baroreflex sensitivity		CZF Fractal variance	no	**	*
**AnyWave** [32]	MATLAB (& C)	Windows, Linux, MacOS	yes [F]	Electrophys-iological signals	.trc, .cnt, .vhdr, .meg4, .bdf, .eeg	no	no	**	**
**ARTiiFACT** [250]	MATLAB	Windows	yes [F]	ECG	ECG, IBI [b]	no	no	***	***
**Biopeaks** [251]	Python		yes [F]	ECG, PPG, respiration	EDF, .txt, .csv, .tsv [b]	no	no	*	
**Biopsychology Nonlinear Analysis Toolbox** [5]	MATLAB		yes [F]	EEG, MEG	ASCII	D2, H, LLE, time-delayed MI		*	*
**BioSig** [252]	C/C++, MATLAB (or Octave)		no [F]	EEG, ECoG, ECG, EOG, EMG, Resp, etc.		no	no	******	***

**BioSPPy** [253]	Python		no [F]	BVP, ECG, EEG, EDA, EMG, Resp, SCG		no	no	***	
**Brainstorm** [19]	MATLAB		yes [F]	EEG, MEG	various [B]			****	****
**CardioSeries** [61]	LabView	Windows	yes [F]	ECG	Txt	no	no	***	*
**Chaos Data Analyzer** [29]	PowerBASIC		no	nonspecific	ASCII [B}	D2, LLE, PP; Hausdorff dimension	no	*	*
**Complexity** [28]	MATLAB		[F]	fMRI		no	ApEn, Cross-ApEn, SampEn		
**EasieRR** [56]	MATLAB	Windows	yes [F]	ECG	ECG (txt, mat)	PP		*	*
**ECG signal acquisition system** [6]	MATLAB		yes	data acquisition & analysis		no	no	**	**
**ECGLab** [57]	MATLAB		no	HRV	ASCII or ECG	PP	no	***	**
**EEGFrame** [20]	Java		yes	EEG	txt, EDF	FD & HFD, AF, H, D2, DFA, LLE, RPA, PP, LZC; Central Tendency Measure (CTM), Sequential Trend Analysis, Spatial Filling Index	CarnapEntropy 1D, CCE (Shannon), MaxApEn, RE, MaxSampEn, Mutual dimension	***	*
**EEGLab** [17]	MATLAB		Yes [F]	EEG	various [B]	no	short-term Rényi entropy [toolbox plug-in]	*	*
**ELAN** [18]	MATLAB (& C)	Windows, Linux, MacOS	yes [F]	EEG, MEG, ECoG, LFP	[B]	no	no	***	**

**EZ Entropy** [9]	MATLAB	Windows, Linux, MacOS	yes [F]	ASCII, .mat, EDF, GDF	ASCII [B]	no	ApEn, SampEn, FE; CE; PE; DistEn	*	**
**FieldTrip** [254]	MATLAB		no [F]	MEG, EEG	various [B]	no	transfer entropy	******	*****
**FracLab** [255]	MATLAB		yes [F]	Fractal analysis		fractal and multifractal analysis: dimensions, Holder exponents, multifractal spectra	no	****	*
**gHRV** [7]	Python	Windows, Linux, MacOS	[F]	HRV	txt, hrm, sdf, ste	FD, PP	ApEn	***	*
**gVARVI** [256]	Python	Windows, Linux, MacOS	yes	HRV		no	no	**	*
**hrv** [58]	Python		yes [F]	HRV	RRi	PP	no	*	*
**HRV Toolkit** (PhysioNet) [257]	C	Windows, Linux, MacOS	no [F]	HRV	txt, wfdb [B]	0	0	***	*
**HRVAnalysis** [15]	MATLAB	Windows	yes [F]	HRV	edf, ishne, binary, txt, mat [B]	HFD, KFD, H, DFA, 1/f slope, LLE, PP, LZC	SE, CE, CCE, normalised CCE, ApEn, SampEn	******	**
**HRVFrame** [14]	Java	Windows, Linux, MacOS	yes [F]	HRV	Txt [B]	FD & HFD, AF, H, D2, DFA, LLE, RPA, PP, LZC; Central Tendency Measure (CTM), Sequential Trend Analysis, Spatial Filling Index	Carnap Entropy 1D,* CCE, fApEn, RE, SampEn	**	*

**HRVAS** [21]	MATLAB		yes [F]	HRV	ibi, txt	DFA, PP	SampEn	***	*
**HRVTool** [47]	MATLAB	Windows, Linux, MacOS	yes [F]	HRV	ECG, IBI; many formats (e.g. EDF, ISHNE, WAV, HRM) [B]	D2, DFA, PP	ApEn	**	*
**Information Breakdown ToolBox** [258]	MATLAB (& C)	Windows, Linux, MacOS	[F]	EEG, local field potentials (LFP)		no	noise/response entropy; mutual information	***	***
**Kaplan's software for HRV** [52]	MATLAB		no	HRV		DFA	ApEn	**	
**KARDIA** [26]	MATLAB	Windows, Linux, MacOS	yes [F]	HRV	IBI [B]	DFA	no	**	**
**Kubios HRV** [13]	MATLAB	Windows, Linux	yes [f]	HRV	IBI, ECG (txt, acq, edf, gdf, dat, hrm, sdf, ste, mat) [b]	D2, RQA, PP	ApEn, SampEn	******	****
**LabVIEW** [4]			yes	signal acquisition & processing		no	no		
**MATLAB-based GUI** [8]	MATLAB		yes			LLE	SE ('entropy')	*	*
**Matrix of Lags (MoL)** [34]				EEG, ECG		FD, DFA, LZC	SE, TE, SampEn	*	*
**MATS** [31]	MATLAB		yes [F]	any scalar time series; EEG	ASCII, .xls, .mat, .edf [B]	D2, Correlation sum, DFA, LZC	ApEn	**	*

**MULTISAB** [33]	Java		yes	ECG, EEG, EMG, …	.an n, .csv, .txt, .edf/.edf+c [B]	as for HRVFrame, plus synchronisation likelihood	as for HRVFrame, plus alphabet entropy *	**	*
**NeuroKit2** [259]	Python		no [F]	ECG, EDA, EMG, EOG, PPG (and eventually EEG)				**	
**Physiolyze (PyHRV)** [43]	Python		yes (web) [F]	HRV	RRi, ECG, BVP	FD, D2, H, DFA, LLE, RQA, PP	ApEn, SampEn, SVD (singular value decomposition) En	**	*
**PhysioNet Cardiovascular Signal Toolbox** [51]	MATLAB	Windows, Linux, MacOS	no [F]	HRV	RRi, ECG (txt, wfdb, mat) [B]	DFA	ApEn, SampEn	***	**
**PhysioScripts** [260]	R	Windows, Linux, MacOS	yes [F]	ECG, RRi, Resp	.txt, .csv [B]	no	no	**	**
**POLYAN** [12]	MATLAB	Windows	yes	heart period, aBP, lung volume, O2 sat., LVV, muscle sympathetic nerve activity		no	no	**	**
**PyBioS** [24]	Python	Windows, Linux, MacOS	yes [F]	cardiovascular (or e.g. Resp, Stride, EEG)	ASCII, .xls or .xlsx [B]	DFA	SampEn, FE, PE, DE, DistEn, PhEn	*	*
**PyEntropy** [261, 262]	Python			analysis of neural data		no	maximum entropy, noise entropy, response entropy	**	**

**PyHRV** [27]	Python	Windows, Linux, MacOS	no [F]	HRV	ECG [B]	DFA, PP	SampEn	**	
**RHRV** [22, 23]	R	Windows, Linux	yes [F]	HRV	ascii, edf+ [B]	generalised D2, Information dimension, DFA, LLE, RQA, PP	ApEn, SampEn	*	*
**RR-APET** [10]	Python	Windows, Linux, MacOS	yes [F]	HRV	txt, mat, h5, wfdb [B]	DFA, RQA, PP	no	**	*
**SinusCor** [64]	MATLAB		yes [F]	HRV	RRi, ECG (.txt, .hrm, .sdf, .bin, .acq, .abf)	PP	no	***	**
**Software tool for HRV** [59]	MATLAB		yes [F]	HRV	ECG, RRi (binary, text or system-specific)	PP (for T-wave alternans, TWA)			
**SPINE-HRV** [263]				HRV	RRi	n	n	**	*
**STAToolkit** [87]	MATLAB (& C)	Windows, Linux, MacOS	no [F]	spike train analysis	txt & other	no	classical & other entropy estimators	**	
**TISEAN** [30]			no [F]			D2, LLE, RQA, PP		******	****
**UW DigiScope** [11]	MATLAB	Windows, Linux, MacOS	yes	signal processing	ECG, EEG, EMG	no	no	*	*
**ViewHRV** [50]	Python		web GUI [F]	HRV		DFA, PP	no	*	*
**WinEEG** [16]			GUI	EEG	ASCII, EDF [B]	no	no	*****	*
